# Practical Notes on Diagnosis and Treatment

**Published:** 1908-04-25

**Authors:** 


### PRACTICAL NOTES ON DIAGNOSIS AND TREATMENT.

**Intermittent Glycosuria.**

Trypsin in 2-grain doses administered thrice daily about three hours after a meal has proved successful in the treatment of intermittent glycosuria.—*Dr. L. A. Clutterbuck.*

**Worms and Appendicitis.**

Cases have been recorded in which thread worms have been found in the mucous membrane of the excised appendix, and attacks of appendicitis have been attributed to their presence.

**Laryngeal Growths.**

The probatory intralaryngeal removal of a part of a suspected growth ought not to be undertaken until after the patient has formally consented to immediate radical operation in the event of the microscope proving the malignancy of the tumour.—*Dr. David Newman.*

**Calcium Chloride.**

The disagreeable taste of this salt may be concealed by saccharine; one minim of the elixir is sufficient to cover the taste of 10 grains of calcium chloride. Sir Lauder Brunton has used calcium salts both in pneumonia and in heart disease to prevent cardiac failure.

**Migraine.**

Given in the very early stage of the attack, a full dose of butyl chloral hydrate and phenazone will often abort the headache; in the same circumstances nitro-glycerine will sometimes prove successful. Between the attacks the three bromides—potassium, sodium, ammonium—may be ordered. In some cases the attacks cease when the patient is kept to a vegetarian diet.

**White Wine Whey in Infant Feeding.**

In cases where, owing to difficulty of milk digestion, there has been chronic vomiting and the infant has become feeble and exhausted, feeding for two or three days on sherry whey alone, and then on alternate feeds of sherry whey and fully peptonised milk, is sometimes of great benefit. To prepare sherry whey: Heat 10 ounces of milk just to the boiling point, then add 2½ ounces of cooking sherry, again heat until the mixture begins to "boil up," allow to stand for three minutes and strain off the curd.—*Dr. Myers and Dr. Still.*

**Static Electricity in Sciatica.**

By this method, sciatica and other forms of neuritis or neuralgia can be rapidly cured if the patient is not too advanced in years and provided he is seen at the outset.—*Dr. J. Curtis Webb.*

**Pyrexia in Cancer.**

Nearly two-thirds of the cases of malignant disease of the liver show some degree of pyrexia, at any rate in their later stages. It is not uncommon to observe successive periods of fever alternating with apyrexial intervals.—*Dr. Jas. W. Russell.*

**Rectal Feeding.**

That patients feel, and often are, better for a time on rectal feeding is due to the fact that the gastric condition which led to the adoption of this method has improved, in consequence of the physiological rest given to the stomach. The belief that rectal feeding can maintain a nutritive equilibrium is a mistaken one.

**Angina Pectoris.**

Fiessenger recommends 5 minims of a 1-in-1,000 solution of crystallised digitalin once daily, and 8 grains of theobromine twice daily—the latter to neutralise the constricting action of the digitalis on the arterioles. He finds this method most successful when the patient has a sense of breathlessness on exertion.

**Pernicious Anæmia.**

There are cases of pernicious anæmia in which the stomach symptoms are the most prominent—pain, sickness, vomiting, sometimes of blood, and gradual loss of weight. These cases so closely simulate ulcerating cancer of the stomach without a palpable tumour that I defy anyone to make a definite diagnosis without a careful examination of the blood.—*Dr. G. Lovell Gulland.*

**Sodium Citrate in Dyspepsia.**

Sodium citrate is useful in dyspepsia in adults, especially when this is accompanied by gastric pain occurring some hours after food and accompanied by vomiting. A tablespoonful of a ten-per-cent. solution should be taken as soon as the symptoms commence and should be repeated every five minutes until they cease. A total of 30 to 60 grains is usually successful.

